# Proper QRS for EMBLEM S-ICD Across Micra Recipients—Pilot Study

**DOI:** 10.3390/jcm14051420

**Published:** 2025-02-20

**Authors:** Bruno Hrymniak, Przemysław Skoczyński, Bartosz Skonieczny, Bartosz Biel, Krystian Josiak, Patrycja Aktanorowicz, Tomasz Wieczorek, Dorota Zyśko, Waldemar Banasiak, Dariusz Jagielski

**Affiliations:** 1 Department of Cardiology, Center for Heart Diseases, 4th Military Hospital, 53-114 Wroclaw, Poland; bruno.hrymniak@gmail.com (B.H.);; 2 Department of Emergency Medicine, Wroclaw Medical University, 50-367 Wroclaw, Poland; 3 Faculty of Medicine, Wroclaw Medical University, 50-367 Wroclaw, Poland; 4 Faculty of Medicine, Wroclaw University of Science and Technology, 50-370 Wroclaw, Poland

**Keywords:** subcutaneous implantable cardioverter-defibrillator, sudden cardiac arrest, sudden cardiac death, S-ICD, Micra, leadless pacemaker, screening

## Abstract

**Background:** In total, 6.6% to 11% of patients with an initially implanted implantable cardioverter-defibrillator (ICD) will develop significant bradycardia and the need for pacing in subsequent years. As the leadless pacemaker (LP) and subcutaneous implantable cardioverter-defibrillator (S-ICD) population comorbidities are often similar, both groups would benefit from a hybrid solution. Unfortunately, currently, there is no commercially available and sufficiently validated interconnected set of S-ICD and LP. **Methods:** In this single-center, prospective observational study, 32 pacing-dependent patients after implantation of a Micra LP were screened for S-ICD on the left and right sides of the sternum using the EMBLEM Automated Screening Tool. At least one positive, both in the supine and standing positions, was considered a positive screening. The impact of various clinical variables and morphology of paced QRS on screening results was assessed. Moreover, the function of the tricuspid valve was evaluated before and after LP implantation to consider whether there is a relationship between paced QRS and worsening tricuspid regurgitation. **Results:** Patients with paced heart rhythm were divided into two groups based on screening results for S-ICD. The positive screening outcome was achieved in 10 patients (31.25%). No correlation between any clinical variable and screening results was found. However, right axis deviation [RAD] of paced QRS seems to be a strong predictor of positive S-ICD screening (RAD in 9/32 patients, sensitivity 90%, specificity 100%, PPV 100%, NPV 96% for passing screening), and negative polarity of paced QRS in inferior leads predicts negative screening results (positive polarity in II, III, and aVF in 12/32 patients, sensitivity 100%, specificity 90%, PPV 83%, NPV 100% for passing screening). **Conclusions:** Right axis deviation of the paced rhythm, positive QRS polarity of leads II, III, and aVF, and negative QRS polarity in leads I and aVL seem to predict a positive screening result for S-ICD. Such a position of LP does not seem to worsen tricuspid regurgitation.

## 1. Introduction

Extravascular cardiac implantable electronic device (CIED) technology overcomes many obstacles and risks of transvenous leads implantation, i.e., the need for subclavian venous access or infective endocarditis (IE) and its sequelae, such as the need for transvenous lead extraction (TLE) [1,2]. It should come as no surprise that the number of implanted devices such as S-ICD or LP is more than 140,000 and 200,000, respectively, and the numbers are still rising.

Unfortunately, such a technology has its limitations. For example, despite the undeniable safety properties of S-ICD, the extravascular electrode leads to a lack of continuous cardiac pacing properties (except for post-shock 30 s of trans-transthoracic ventricular pacing) or intracardiac electrogram sensing.

Moreover, the number of staff needed for implantation of the S-ICD is usually greater, and it is performed by cardiologists in cooperation with an anesthesiologist due to the recommended defibrillation test after implantation and the painful nature of the procedure. The methods of anesthesia depend on the risk of personal procedures and patient status. New methods of multimodal anesthesia, such as fascial anesthesia, allow for increased accessibility to these specific treatments [3].

Lack of intracardiac electrograms increases the probability of inappropriate therapies (IATs) among S-ICD recipients due to T-wave oversensing [4]. Thus, a positive screening for S-ICD is essential before implantation.

Fortunately, 85.8% of patients with intrinsic rhythm pass the screening tests [5]. Every patient who fails screening for the standard left-sided electrode location of S-ICD should have another screening on the right side of the sternum [6]. Such a maneuver further increases the percentage of positive screening results among the population with intrinsic rhythm [2].

Due to the mentioned absence of the continuous cardiac pacing properties of S-ICD, among the inclusion criteria for such a device is the lack of a need for it. However, 6.6% to 11% of patients with an initially implanted ICD will have indications for pacing in subsequent years [3,4].

In turn, LP overcomes the shortcomings of S-ICD pacing properties and the need for a larger implantation team; however, it does not prevent sudden cardiac death.

The ideal hybrid solution for such a group needing SCD prevention and cardiac pacing would be the cooperation of S-ICD and LP.

Unfortunately, currently, there is no commercially available and sufficiently validated interconnected set of S-ICD and LP or other cardiac implantable electronic devices. However, such a solution is currently under development and will soon be available as another LP option, providing the unique possibility of antitachycardia pacing (ATP) [7]. Moreover, the automatic screening tool for S-ICD has not been validated for paced rhythms.

## 2. Aims

The study aims to assess the possibility of positive screening among the EMBLEM S-ICD across Micra recipients. Moreover, the study tries to determine whether the paced heart axis has an impact on the positive screening frequency and describes factors that might favor positive S-ICD screening in Micra recipients.

## 3. Materials and Methods

This is a single-center, observational pilot study conducted in a Cardiology Department in Poland among patients with an implanted Micra VR or AV between January 2020 and June 2024. Only pace-dependent Micra recipients were included in the study.

Every venous access for an LP was achieved from the femoral vein. The septal LP position was assured with available radiological signs and afterward with postprocedural transthoracic echocardiography (TTE) [8]. Moreover, postprocedural TTE also evaluated any worsening of the tricuspid valve regurgitation.

The screening for the S-ICD was conducted between October 2023 and June 2024 using the Emblem Automated Screening Tool from LATITUDE™ Programming System (Boston Scientific, Marlborough, MA, USA), along with the producer instructions, with ongoing ventricular stimulation at 70 bpm. The left arm (LA) electrode was placed 1.5 cm to the left from the xiphoid, the right arm (RA) electrode 14 cm upward from LA, and the left leg (LL) electrode was in the line of the anterior armpit line parallel to the LA electrode. A sensing check for S-ICD vectors was performed on the left and right sides of the sternum. At least one positive, both in the supine and standing positions, was considered a positive screening.

The screening result and the reason for the eventual sensing failure were noted.

A standard 12-lead-ECG was obtained after the screening. The QRS polarity was assessed via paced QRS net amplitude.

The paced heart rhythm axis was evaluated as follows:

- Normal Axis: QRS net amplitude positive in leads I and aVF or positive in leads I and II and negative in lead aVF
- Left Axis: QRS net amplitude positive in lead I and negative in leads II and aVF
- Right Axis: QRS net amplitude negative in lead I and positive in lead aVF
- Extreme Axis: QRS net amplitude negative in leads I and aVF

**Figure 1:**
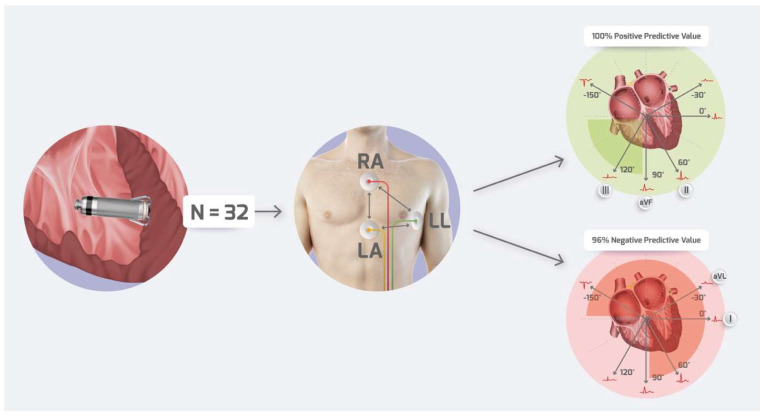
Graphical abstract of the pilot study. RA—right arm, LA—left arm, LL—left leg.

Patients were divided into two groups with negative and positive screening outcomes for S-ICD. The pilot-study design summary is presented in Figure 1. Patients’ data were anonymized, combined into a database, and statistically analyzed as a cohort. The institutional review board approved the study protocol. The patients’ data were protected according to national law requirements and hospital standard operating procedures.

All patients gave informed consent to participate in the study. The study was conducted in accordance with the Declaration of Helsinki.

## 4. Statistical Analysis

The results are presented as mean ± SD, or as number and percentage for continuous and discrete variables. The distribution of continuous variables was checked with the Shapiro–Wilk test to determine the normality of the data. The Mann–Whitney test (unpaired data, two-tailed) was used for non-normally distributed data, and an unpaired *t*-test was used to compare normally distributed data groups. The chi-square test with Yates correction and Fischer’s exact test were used to assess the significance of differences between discrete variables. Negative and positive predictive values for positive S-ICD screening were calculated. A *p*-value < 0.05 was considered statistically significant. All data analyses were performed using Statistica 13.1 Software (TIBCO Software, Palo Alto, CA, USA).

## 5. Results

Baseline characteristics are shown in Table 1. Ten patients (32%) achieved a positive screening outcome. None of the assessed demographic and clinical variables correlate with screening results (Table 1).

Paced QRS polarity/axis in 12 leads and screening results are summarized in Table 2 and Table 3. The most commonly paced QRS axis was LAD—it was achieved in 53.1% of patients. NAD and READ were the rare axes, present in only 9.1% of patients.

None of the patients with negative QRS polarity in any of the inferior leads passed S-ICD screening (positive polarity in II, III, and aVF in 12/32 patients, sensitivity 100%, specificity 90%, PPV 83%, NPV 100% for passing screening). Similarly, none of the patients with paced QRS LAD passed S-ICD screening. On the other hand, every patient with RAD had positive screening results (RAD in 9/32 patients, sensitivity 90%, specificity 100%, PPV 100%, NPV 96% for passing screening).

90% of patients in the positively screened group had a right axis deviation (RAD) during paced rhythm, while no patient in the negatively screened group had such a heart axis (Table 3). Thus, RAD seems to have the strongest positive predictive value (PPV) and negative predictive value (NPV) for passing screening for S-ICD. In turn, left axis deviation (LAD) or right extreme axis deviation (READ) of the heart was present in 90.1% of negatively screened patients with paced rhythm. None of the patients with READ or LAD were positively screened for S-ICD.

Positive QRS polarity in leads II (*p* < 0.001), III (*p* < 0.001), and aVF (*p* < 0.001), and negative polarity in a lead aVL (*p* < 0.001) and I (*p* < 0.001) achieved statistical significance (Table 2) for positive screening results. There was no worsening of tricuspid valve regurgitation in either of the groups.

## 6. Discussion

Several studies have evaluated automatic screening for S-ICD results in paced rhythms [9,10]. However, they did not include LP recipients. Since LP pacing rhythm resembles classical septal or apical stimulation, such screening results should be compared. In a patient group with classical right ventricle septal stimulation described by Opielowska-Nowak B. et al., 25.4% of patients underwent positive screening results [9]. Our observation showed a higher percentage of positive screening results in our population with LP than in classical transvenous pacing. Importantly, in the above-mentioned study, there was no evaluation of QRS polarity and heart-paced axis impacting screening results.

In the early era of leadless pacing, the right ventricle apex used to be a favorable location for Micra implantation. Such an LP position makes obtaining positive QRS polarity in II, III, and aVF leads impossible through stimulation [11]. Thus, such a stimulation seems to be an unfavorable place for LP positioning regarding potential future S-ICD implantation. Fortunately, currently, there is no such recommendation for the Micra system.

Moreover, the percentage of positive screening results can differ between the delivered LP due to the LP measurements and catheter maneuverability. Such nuanced differences can lead to different achievable septal positions. That said, the apical part of the septum is often the only available implantation site for Aveir PM (Abbott, Abbott Park, IL, USA) due to its length (38 mm) and, therefore, more difficult maneuverability. It may result in a greater risk of screening failure for S-ICD across Aveir recipients.

The expected QRS polarity configuration (positive QRS in II, III, and aVF leads and negative in I and aVL) and paced heart axis for positive S-ICD screening test occur in mid-septum and right ventricular outflow tract septal stimulation [11]. Nonetheless, such an LP position poses the hypothetical risk of the tricuspid valve impact worsening the regurgitation due to the shorter distance between the mid-septum and the tricuspid valve than the apex-tricuspid valve. However, no worsening regurgitation was observed in our post-procedure evaluation with TTE. The conclusions of research conducted by Haeberlin A. et al. are similar [12]. In their results, the prevalence of worsening tricuspid regurgitation after LP did not achieve statistical significance.

In agreement with Lewandowski M. et al., every patient screened for S-ICD should be tested on both left and right sternal locations. This allows, among other things, for achieving positive screening results even if standard position-sensing electrodes fail [6]. However, in our observation, two patients passed the screening test on the right side of the sternum. One passed screening both via the left side of the sternum and the other only on the right part of the sternum.

It is worth noting that the study group consisted of patients with pacing dependency. This is a convenient situation during patient qualification for screening because the Boston S-ICD does not change sensing vectors during alterations between intrinsic and paced rhythm. However, the circumstances can be perilous if the positive sensing vectors of intrinsic and paced rhythm differ.

Since venous access from the jugular vein became available and more popular for Micra implantation [13], the question has arisen regarding whether such implantation has any implication for the septal position of Micra, i.e., with more favorable QRS polarity for S-ICD screening. Further studies should be conducted.

Micra was not designed to co-work with S-ICD and its high-voltage therapy. However, along with Micra’s VR and AV instructions, it can withstand electrical shock–defibrillation, or cardioversion with paddles or patches. Nonetheless, several precautions must be made, such as using the lowest clinically appropriate energy or positioning the paddles at least 15 cm from the implanted Micra [8]. We performed one defibrillation test after Micra VR and S-ICD hybrid implantation in our clinic. No issues were noted after the procedure—the Micra threshold and impedance were comparable before and after the shock. Moreover, S-ICD was still capable of sensing paced QRS correctly. During one year of follow-up, there were no reported IATs. Similar experiences with no IATs were also observed in another case in Poland [14]. Currently, pacing-dependent patients who have previously undergone Micra implantation and require protection against sudden cardiac death are being prepared for EMBLEM S-ICD implantation. All of them have passed the screening for EMBLEM S-ICD.

Finally, the EMPOWER LP (Boston Scientific, Marlborough, MA, USA) will soon be available, offering full integration with the EMBLEM S-ICD, including ATP delivery and pacing. The MODULAR-ATP study confirmed that combining an EMPOWER LP with an EMBLEM S-ICD is a safe and effective approach, providing both pacing and protection against sudden cardiac death [7]. Notably, this modular system achieved a 98.8% success rate in wireless communication, and ATP therapy from EMPOWER LP successfully terminated 61.3% of arrhythmias.

However, the impact of coexisting LPs in the right ventricle on successful S-ICD communication with EMPOWER LP remains unknown. To date, the simultaneous existence of two LPs implanted in the right ventricle has been reported, with the potential for the presence of up to three [8].

Furthermore, although ATP therapy is an important method for terminating ventricular arrhythmias, patients with an EMBLEM S-ICD did not have such an option until the MODULAR-ATP trial and arrival of EMPOWER LP. Nevertheless, they have still benefited successfully from S-ICD. Thus, it raises the question of whether patients with a previously implanted Micra should also receive an EMPOWER LP.

## 7. Limitations

The study’s main limitation was the small sample size.

Automated S-ICD screening has not been validated for paced rhythms. Thus, a positive screening result does not necessarily mean proper cooperation with the tested devices.

Moreover, a positive screening result does not necessarily mean proper arrhythmia detection. Thus, a defibrillation test is still necessary after the S-ICD implantation procedure.

Moreover, the sensing process was performed at a fixed ventricular rate, and the outcome of the sensing process could not be predicted during pacing at a faster rate.

## 8. Conclusions

Positive S-ICD screening for pacing-dependent patients after Micra implantation is achievable. However, in this study, it was observed in only 31% of screened patients.

A right axis deviation and an inferior axis of the paced heart rhythm were the strongest predictors of a positive screening result. Therefore, in patients with a long life expectancy who qualify for leadless pacemaker implantation, pace-mapping during the procedure should be considered to optimize QRS polarity and improve the likelihood of a successful S-ICD screening. The ideal target position for a leadless pacemaker in these patients appears to be at least mid-septal or higher in the right ventricle.

## Figures and Tables

**Table 1:** Patient characteristics and clinical presentation.

Variable	Negative Screening (n = 22)	Positive Screening (n = 10)	*p*-Value
Male gender	13 (59.1%)	4 (40%)	0.53
Age [years]	75.09 ± 15.22	73.2 ± 19.41	0.98
BMI [kg/m^2^]	26.62 ± 3.26	24.5 ± 3.51	0.20
Anemia	8 (36.4%)	5 (50%)	0.73
Thrombocytopenia	3 (13.6%)	1 (10%)	0.77
Kidney failure	6 (27.3%)	4 (40%)	0.76
Diabetes mellitus	8 (36.4%)	3 (30%)	0.96
Previous transvenous lead extraction due to infection	5 (22.7%)	1 (10%)	0.71
Previous transvenous lead extraction due to lead damage	3 (13.6%)	2 (20%)	0.94
Lead dysfunction	3 (13.6%)	3 (30%)	0.54
Heart Failure	2 (9.1%)	1 (10%)	0.57
Ejection Fraction (%)	52.73 ± 7.36	53.5 ± 8.83	0.55
Coronary artery disease	4 (18.2%)	2 (20%)	0.71
Previous myocardial infarction	3 (13.6%)	2 (20%)	0.95
Previous percutaneous Coronary intervention	3 (13.6%)	1 (10%)	0.77
Previous coronary artery bypass grafting	0 (0%)	0 (0%)	
Hypertension	17 (77.3%)	6 (60%)	0.56
Atrial fibrillation	10 (45.5%)	5 (50%)	0.89
Previous transcatheter aortic valve implantation	1 (4.5%)	1 (10%)	0.84
Previous atrial valve replacement	4 (18.2%)	1 (10%)	0.95
Previous tricuspid valve replacement	1 (4.5%)	1 (10%)	0.84
Neoplasm	1 (6.3%)	2 (28.6%)	0.46
Worsening tricuspid valve regurgitation after Micra implantation	0 (0%)	0 (0%)	

**Table 2:** Paced QRS polarity QRS axis and screening results.

Polarity/Axis	Screening (+) n = 10	Screening (−) n = 22	Total n = 32
I +	1 (10%)	18 (81.8%)	19 (59.4%)
II +	10 (100%)	2 (9.1%)	12 (37.5%)
III +	10 (100%)	4 (18.2%)	14 (43.8%)
AVL +	0 (%)	19 (86.4%)	19 (59.4%)
AVF +	10 (100%)	3 (13.6%)	13 (40.6%)
AVR +	2 (20%)	12 (54.5%)	14 (43.8%)
V1 +	0 (%)	7 (31.8%)	7 (21.9%)
V2 +	0 (%)	2 (9.1%)	2 (6.3%)
V3 +	0 (%)	1 (4.5%)	1 (3.1%)
V4 +	1 (10%)	1 (4.5%)	2 (6.3%)
V5 +	4 (40%)	2 (9.1%)	6 (18.8%)
V6 +	5 (50%)	4 (18.2%)	9 (28.1%)
RAD	9 (90%)	0 (0%)	9 (28.1%)
LAD	0 (0%)	17 (77.3%)	17 (53.1%)
NAD	1 (10%)	2 (9.1%)	3 (9.4%)
READ	0 (%)	3 (13.6%)	3 (9.4%)

**Table 3:** Specificity, sensitivity, and positive and negative predictive values of paced heart polarity and axis for positive S-ICD screening result.

Positive Polarity/ Axis	Yes, Positive Screening	Yes, Negative Screening	No, Negative Screening	No, Positive Screening	Sensitivity	Specificity	PPV	NPV
I +	1	18	4	9	10.00	18.18	5.26	30.77
II +	10	2	20	0	100.00	90.91	83.33	100.00
III +	10	4	18	0	100.00	81.82	71.43	100.00
AVR +	2	12	10	8	20.00	45.45	14.29	55.56
AVL +	0	19	3	10	0.00	13.64	0.00	23.08
AVF +	10	3	19	0	100.00	86.36	76.92	100.00
V1 +	0	7	15	10	0.00	68.18	0.00	60.00
V2 +	0	2	20	10	0.00	90.91	0.00	66.67
V3 +	0	1	21	10	0.00	95.45	0.00	67.74
V4 +	1	1	21	9	10.00	95.45	50.00	70.00
V5 +	4	2	20	6	40.00	90.91	66.67	76.92
V6 +	5	4	18	5	50.00	81.82	55.56	78.26
RAD	9	0	22	1	90.00	100.00	100.00	95.65
LAD	0	17	5	10	0.00	22.73	0.00	33.33
NAD	1	2	20	9	10.00	90.91	33.33	68.97
READ	0	3	19	10	0.00	86.36	0.00	65.52
II, III, aVF +	10	2	20	0	100.00	90.91	83.33	100.00

## Data Availability

All data are archived at the Cardiology Clinic in 4th Military Hospital, Wroclaw, Poland and can be provided by the author.

## Abbreviations

The following abbreviations are used in this manuscript:

ATP—antitachycardia pacing
CIED—cardiovascular implantable electronic device
IAT—inappropriate therapy
IE—infective endocarditis
LAD—left axis deviation
LP—leadless pacemaker
NAD—normal axis deviation
RAD—right axis deviation
READ—right-extreme axis deviation
S-ICD—subcutaneous implantable cardioverter-defibrillator
TLE—transvenous lead extraction
TTE—transthoracic echocardiography

## References

[1] Bongiorni M.G., Kennergren C., Butter C., Deharo J.C., Kutarski A., Rinaldi C., Romano S.L., Maggioni A.P., Andarala M., Auricchio A. (2017). The European Lead Extraction ConTRolled (ELECTRa) study: A European Heart Rhythm Association (EHRA) Registry of Transvenous Lead Extraction Outcomes. Eur. Heart J..

[2] Blomström-Lundqvist C., Traykov V., Erba P.A., Burri H., Nielsen J.C., Bongiorni M.G., Poole J., Boriani G., Costa R., Deharo J.-C. (2020). European Heart Rhythm Association (EHRA) international consensus document on how to prevent, diagnose, and treat cardiac implantable electronic device infections—Endorsed by the Heart Rhythm Society (HRS), the Asia Pacific Heart Rhythm Society (APHRS), the Latin American Heart Rhythm Society (LAHRS), International Society for Cardiovascular Infectious Diseases (ISCVID), and the European Society of Clinical Microbiology and Infectious Diseases (ESCMID) in collaboration with the European Association for Cardio-Thoracic Surgery (EACTS). Eur. Heart J..

[3] Szamborski M., Janc J., Leśnik P., Milnerowicz A., Jagielski D., Łysenko L. (2023). Regional Anesthesia for the Implantation of a Subcutaneous Implantable Cardioverter-Defibrillator Using Pectoserratus Plane Block and Superficial Serratus Anterior Plane Block. Med. Sci. Monit..

[4] El-Chami M.F., Harbieh B., Levy M., Leon A.R., Merchant F.M. (2016). Clinical and electrocardiographic predictors of T wave oversensing in patients with subcutaneous ICD. J. Arrhythmia.

[5] Gold M.R., El-Chami M.F., Burke M.C., Upadhyay G.A., Niebauer M.J., Prutkin J.M., Herre J.M., Kutalek S., Dinerman J.L., Knight B.P. (2023). Postapproval Study of a Subcutaneous Implantable Cardioverter-Defibrillator System. J. Am. Coll. Cardiol..

[6] Lewandowski M., Syska P., Kowalik I. (2023). Standard and extended electrocardiographic screening for the subcutaneous implantable cardioverter-defibrillator. Europace.

[7] Knops R.E., Lloyd M.S., Roberts P.R., Wright D.J., Boersma L.V., Doshi R., Friedman P.A., Neuzil P., Blomström-Lundqvist C., Bongiorni M.G. (2024). A Modular Communicative Leadless Pacing–Defibrillator System. N. Engl. J. Med..

[8] Hrymniak B.M., Skoczyński P., Biel B., Banasiak W., Jagielski D. (2023). Atrioventricular synchronous leadless pacing: Micra AV. Cardiol. J..

[9] Opielowska-Nowak B., Kempa M., Budrejko S., Sławiński G., Raczak G. (2022). Eligibility of patients with temporary paced rhythm for a subcutaneous implantable cardioverter-defibrillator. Kardiol. Pol..

[10] Molina-Lerma M., Cabrera-Borrego E., Rivera-Lopez R., Sánchez-Millán P., Mellado J.P., Jiménez A.A., Álvarez M. (2023). Comparison of automated subcutaneous defibrillator screening between different pacing sites in cardiac pacing device carriers. Europace.

[11] Das A., Kahali D. (2018). Ventricular septal pacing: Optimum method to position the lead. Indian Heart J..

[12] Haeberlin A., Bartkowiak J., Brugger N., Tanner H., Wan E., Baldinger S.H., Seiler J., Madaffari A., Thalmann G., Servatius H. (2022). Evolution of tricuspid valve regurgitation after implantation of a leadless pacemaker: A single center experience, systematic review, and meta-analysis. J. Cardiovasc. Electrophysiol..

[13] Saleem-Talib S., van Driel V.J., Nikolic T., van Wessel H., Louman H., Borleffs C.J.W., van der Heijden J., Cox M., Ramanna H. (2022). The jugular approach for leadless pacing: A novel and safe alternative. Pacing Clin. Electrophysiol..

[14] Kaczmarek K., Czarniak B., Jakubowski P., Wranicz J.K., Ptaszyński P. (2018). Leadless pacemaker and subcutaneous implantable cardioverter-defibrillator therapy: The first use of a novel treatment option in Poland. Kardiol. Pol..

